# Editorial: Insights in pediatric urology: 2021

**DOI:** 10.3389/fped.2023.1174553

**Published:** 2023-03-31

**Authors:** Miguel A. Castellan, Lisandro A. Piaggio, Barroso Jr Ubirajara

**Affiliations:** ^1^ Department Pediatric Urology, Nicklaus Children’s Hospital, Joe Di Maggio Children’s Hospital, University of Miami, Miami, FL, United States; ^2^ Department Pediatric Urology, Universidad Nacional del Sur, Bahía Blanca, Argentina; ^3^ Federal University of Bahia, Salvador, Brazil

**Keywords:** hypospadias, hydronephrosis, testicular torsion, circumcision, ureteric, pyeloplasty stones

**Editorial on the Research Topic** Insights in pediatric urology: 2021

The purpose of this research Topic is to highlight the best research topic insights of Pediatric Urology in 2021. In this collection of articles published on this subject, we have a wide selection of studies that will contribute to the further progress of the field. In this issue, authors shared their experience with various urological anomalies and their management accompanied by a literature review. We thank the authors and reviewers for sharing their experiences and making these manuscripts better.

Bar-Yaakov et al. showed that decisional regret was reported by 27.5% of parents of children who underwent revision of neonatal circumcision for excess foreskin. Parents were concerned about the appearance of a penis which may resemble an uncircumcised penis and most parents do not regret the surgery (1). This study is well conducted and gives additional information to discuss with parents before indicating this surgery. However, since a non-therapeutic approach is employed most of the time, caregivers must be provided with sufficient information about the pros and cons of the surgery so that they can make the best decision and avoiding any regret in the future (2).

Authors from Spain (Ordóñez et al.), described the long-term outcome of the patients with ureteropelvic junction obstruction (UPJO) treated by endoscopic retrograde balloon dilatation (ERBD). This is the largest reported case series treated endoscopically in children with primary unilateral UPJO (92 cases being younger than 18 months). An additional procedure was needed in 21% of patients and an open pyeloplasty was needed in 6.25% (*n* = 7) cases of technical failure. Long-term success rate was 76.8% after one dilatation, and 86.6% in those who required up to 2 dilatations. Robotic and laparoscopic pyeloplasty are now frequently done in small children and, this data is very important and useful to discuss with parents before indicating surgery (3). Benefits of minimal invasive surgery and success rate should be balanced with chances of second surgery and additional anesthesia.

Xiaofei and Benzhang report a case of testicular torsion (TT) in a patient with unilateral supernumerary testis (SNT). Since timing at presentation, the twisted gonad in the hemi-scrotum with SNT could not be saved but the remaining uninvolved testicle was preserved. The authors should be congratulated for the surgical criteria and the effort to preserve normal tissue. Intravaginal TT occurred in a clockwise direction in a left testicle, contrary what happens in most cases (inward rotation), probably because testicles are in different planes and there were no scrotal attachments (4). Orchidopexy in the contralateral hemi-scrotum was a wise decision.

The ideal technique for the correction of hypospadias would be one that allows for a good aesthetic and functional result, with a slit urethral meatus, correction of the penile curvature, obtaining an appropriately sized phallus and a low rate of complications. Unfortunately, so far, no technique has reached all these goals (5). Xie et al. compared two surgical techniques for proximal hypospadias, the staged TIP urethroplasty (group 1), and the Byars’ two-stage urethroplasty (group 2). The authors found a significantly lower complication rate in patients in group 1. As it was not a randomized clinical trial, the results of this study should be evaluated with caution, as less severe cases may have been selected to undergo TIP. Furthermore, follow-up is not long enough to detect complications that may occur later such as urethral strictures (5).

Authors from Italy, Paraboschi et al. reported an update on the current management of pediatric urolithiasis. This important review article describes novel technologies and devices to minimize surgical complications and improve patient outcomes. Small instruments and new technological developments have led to many innovations in the surgical management of pediatric urolithiasis (Peng et al.). The devices and technologies described in this review article are changing the way surgeons plan and treat urinary stones in children. Pediatric urologists need to be up to date with these technological advances that can decrease the morbidity of patients with urinary stones.

Cryptorchidism is a common medical condition routinely treated by pediatric urologists. In the case of a non-palpable testis, in around half of the cases no intraabdominal testis will be found. In the presence of a vanishing testis- also known as testicular regression syndrome (TRS), there is a debate whether surgical excision of the remnant is necessary (6). Depending on the presence of ectopic seminiferous tubules or germ cells in the remnant there is potential for malignancy. Gao et al. from China made a research handling more than 300 patients with TRS in which they found very few germ cells in testicular remnants concluding that an inguinal or scrotal exploration may not be necessary. They share good evidence in this paper which is worth reading for any surgeon dealing with this condition. Having this information handy would help in more grounded decision making for this condition.

Bergel et al. from Germany, analyze in their study the safety and outcome of performing pediatric transplant exclusively in a pediatric environment. In their Institution, pediatric renal transplants were performed over a long period in the general urology and high-volume transplant setting. In 2013, the decision was made to change this setting and pediatric transplantations were since then solely carried out in the pediatric hospital setting. Their data and others showed a trend to transplant children at a younger age (7). In this manuscript they described the importance of ensuring a Pediatric medical environment that can address their challenges in perioperative care according to their individual needs.

This Research Topic and all these manuscripts illustrate the current approach to different pediatric urology clinical anomalies and contribute to their management. We are grateful to all who contributed so generously to this effort and hope that readers will enjoy and profit from these articles.
